# From few to many: How non-model organisms’ genomics broadened our understanding of molecular evolution

**DOI:** 10.1590/1678-4685-GMB-2025-0243

**Published:** 2026-07-31

**Authors:** Mariana F. Nery, Beatriz Daros, Ana Luiza Lein-Borba, Iasodara C. L. Santos, Beatriz F. Leite, Luana Nara

**Affiliations:** 1 Universidade Estadual de Campinas, Instituto de Biologia, Departamento de Genética, Evolução, Microbiologia e Imunologia, Campinas, SP, Brazil.

**Keywords:** Comparative genomics, convergent evolution, genome evolution, next-generation sequencing, speciation

## Abstract

For much of the 20th century, our understanding of genetics and evolution was predominantly shaped by intensive studies of a few model organisms such as *Drosophila melanogaster*, *Caenorhabditis elegans*, and *Mus musculus*. While these species provided fundamental insights, their laboratory-adapted characteristics potentially made them evolutionary outliers. The advent of next-generation sequencing technologies and sophisticated bioinformatic tools has increased accessibility of genomic research, enabling comprehensive studies of diverse non-model organisms across the tree of life. This expansion has substantially transformed our understanding of molecular evolution, revealing, for example, that convergent evolution operates through multiple mechanisms across different organizational levels, that speciation is a genomically heterogeneous process involving structural variants and adaptive introgression, and that genome architecture exhibits extensive variation in size, content, and organization. Using aquatic mammals as exemplars, we illustrate how comparative genomics of non-model species illuminates the molecular basis of convergent and divergent adaptations. This paradigm shift demonstrates that understanding evolution’s general principles and creative solutions requires embracing life’s full diversity.

## Introduction

For much of the 20th century, our understanding of genetic mechanisms and evolutionary processes was predominantly shaped by intensive studies of a small set of model organisms. *Drosophila melanogaster*, *Caenorhabditis elegans*, *Mus musculus*, *Arabidopsis thaliana*, and *Escherichia coli* became the foundation upon which modern genetics was built (Davis, 2004). The concentrated effort on these few species yielded important insights, from the discovery of fundamental genetic principles to detailed molecular mechanisms governing development and physiology (Wangler et al., 2015).

However, this model-organism-centric approach, while undeniably powerful, created an implicit assumption that the molecular and evolutionary mechanisms observed in these few species were representative of life’s diversity (Jenner and Wills, 2007). The very features that made these organisms ideal for laboratory work, such as simplified genomes, rapid reproduction, and tolerance of inbreeding, were key reasons for their selection as model systems. However, these same traits already made them exceptions to the general biological rule, limiting the extent to which insights derived from these species can be generalized across evolutionary diversity (Alfred and Baldwin, 2015; Russell et al., 2017).

**Figure 1 -:**
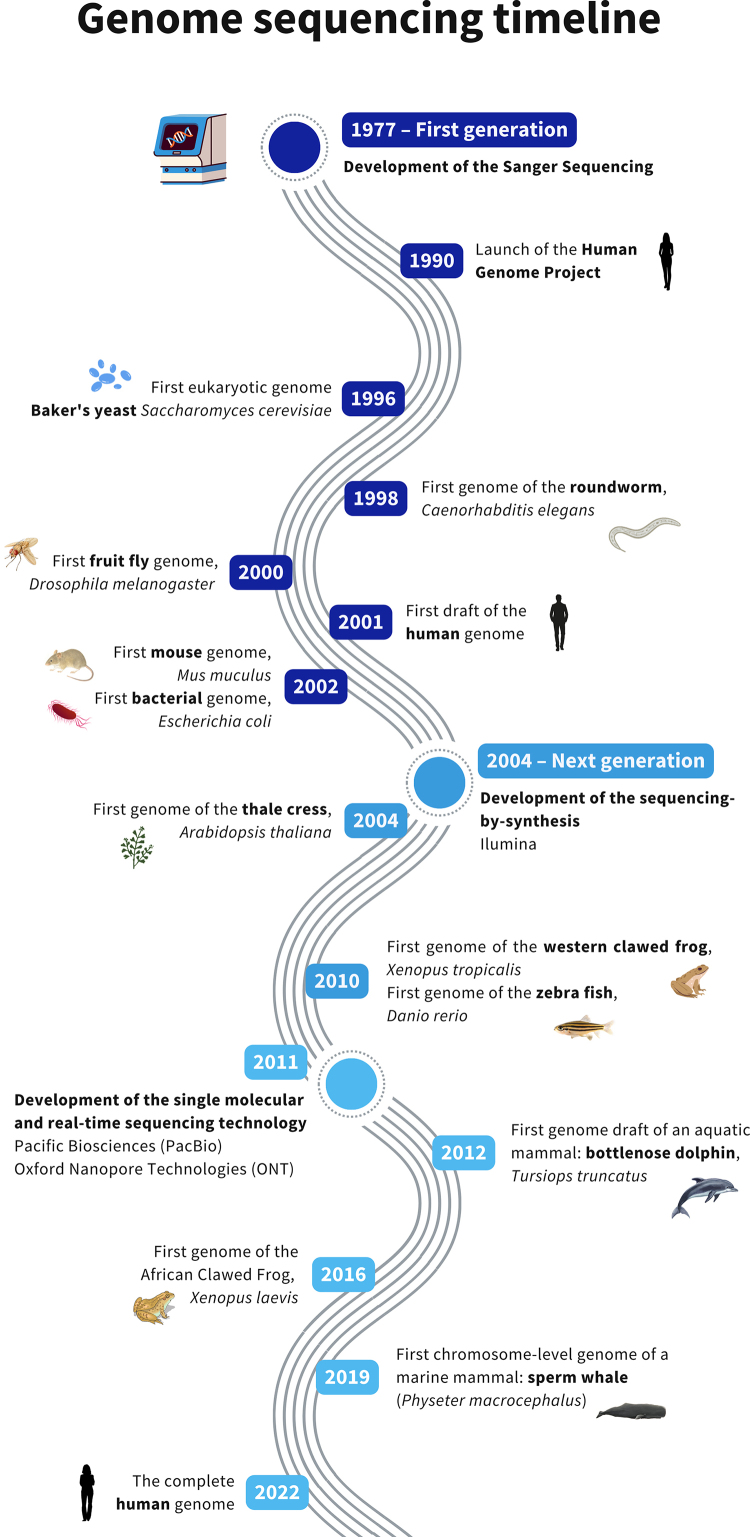
Genome sequencing timeline. The diagram presents the development of sequencing technology in relation to the first model organism genome and the advent of non-model organism genomes.

As we look back at the trajectory of genetic research over the past decades, we can see a great transformation from a field focused on a handful of laboratory-adapted species to a diverse exploration of molecular evolutionary frameworks across the entire tree of life (Ellegren, 2014; Hotaling et al., 2021) (Figure 1). This paradigm shift emerged from the convergence of several factors: growing recognition of the limitations inherent in studying only a few species, technological breakthroughs in DNA sequencing and computational biology, and an increasing appreciation that many fundamental evolutionary questions could only be answered by studying organisms in their ecological and phylogenetic contexts (Abzhanov et al., 2008; Goldstein and King, 2016).

This review examines how the expansion of genomic studies beyond traditional model organisms has transformed our understanding of molecular evolution. We discuss how technological advances have enabled sophisticated studies of non-model species, revealing insights into evolutionary processes and challenging established assumptions. Using examples from various taxa, particularly aquatic mammals, we demonstrate how studying diverse organisms has enhanced our comprehension of convergent evolution, speciation, and the dynamics of genome evolution. 

## The era of model organisms: Foundations and limitations

The concept of ‘model organism’ emerged gradually throughout the 20th century, as geneticists and developmental biologists concentrated their efforts on a selected few species that offered experimental advantages. Thomas Morgan’s choice of *Drosophila melanogaster* in the early 1900s set a precedent that would shape genetic research for decades to come and would become central to the formulation of the chromosome theory of inheritance (Morgan *et al*., 1915). The fruit fly’s rapid life cycle, ease of maintenance, and giant chromosomes made it an ideal subject for classical genetic experiments, and later, studies of mutants uncovered conserved genetic networks that govern development and disease (Lewis, 1955, 1978; Nüsslein-Volhard and Wieschaus, 1980). 

As the field progressed, additional model organisms were established for specific experimental advantages. *Caenorhabditis elegans* combined simplicity with multicellular complexity: its cell lineage is fully traceable (Brenner, 2009), and it has been crucial for elucidating conserved pathways in programmed cell death, aging and disease (Brenner, 1974; Kenyon, 1988; de Bono and Maricq, 2005). Yeast species such as *Saccharomyces cerevisiae* allowed insights into eukaryotic cell biology and genetics in a unicellular system. Pioneering studies using this yeast species identified fundamental regulators of the cell cycle and introduced the concept of checkpoints, providing key insights into cancer biology and genomic stability (Hartwell and McLaughlin, 1968; Hartwell and Weinert, 1989; Nurse, 2002). In prokaryotes, *Escherichia coli* became a cornerstone of molecular genetics after demonstrating genetic recombination (Lederberg and Tatum, 1946), leading to the discovery of DNA polymerase and transcriptional regulation (Jacob and Monod, 1961). It remains vital in recombinant DNA technology (de Marco, 2025) and as a model for chemotaxis (Karmakar, 2021) and pathogenesis (Geurtsen et al., 2022).

More recently, *Danio rerio* emerged as a versatile vertebrate model due to its small size, external fertilization, and high fecundity, supporting studies in development, toxicology, and disease modeling (Grunwald and Eisen, 2002; Varga, 2018). Amphibians such as *Xenopus laevis* and *Xenopus tropicalis* remain powerful model systems in developmental biology and beyond, including toxicology, ethology, and neurobiology (Carotenuto et al., 2023). Among mammals, *Mus musculus* provided a mammalian system with genetic tractability and physiological relevance to humans. It shares many developmental pathways and diseases, and is genetically tractable through embryonic stem cells and homologous recombination (Capecchi, 1989; Standley et al., 2024). Mice have been indispensable for studying cancer (Gercakova et al., 2024), diabetes (Daniels et al., 2021), neurodegenerative disorders (de Bem et al., 2020), thrombosis (Li et al., 2023a) and stem cell biology (Kim et al., 2023). 

In plants, *Arabidopsis thaliana* was established in the 1980s as the leading model, with its compact genome, short life cycle, and extensive genetic resources enabling discoveries in organogenesis, circadian rhythms, immunity, and responses to environmental cues (Meyerowitz and Pruitt, 1985; Yaschenko et al., 2025).

Although classical model organisms have been extremely valuable for elucidating fundamental biological processes, reliance on a restricted set of taxa inherently limits the generalizability of discoveries across the broader tree of life. Most models represent only a few phylogenetic lineages, introducing a strong phylogenetic bias. Critics have also questioned the scientific validity of these models, arguing that their use tends to emphasize the unity of life and genetic mechanisms while neglecting biological diversity and other levels of organismal complexity (Bertile et al., 2023; Kunze and Malfatti, 2025). 

## Technological advances: Expanding access to genetic studies

DNA sequencing technologies have undergone a great transformation since the introduction of the Sanger method in 1977 (Sanger *et al.*, 1977). Subsequent advances in automation and laboratory workflows during the late 20th century enabled increasingly large-scale sequencing efforts, culminating in landmark projects such as the first complete eukaryotic genome, *Saccharomyces cerevisiae* (Goffeau et al., 1996), and the Human Genome Project (International Human Genome Sequencing Consortium, 2001). Over the last two decades, the cost of genome sequencing has dropped dramatically, largely driven by advances in sequencing technologies (Eisenstein, 2023).

The advent of next-generation sequencing (NGS) platforms in the mid-2000s marked a turning point by enabling massively parallel sequencing and greatly reducing both time and cost. Combined with improvements in sequencing chemistry, read lengths, and computational methods for genome assembly and annotation, these advances expanded access to genomic data and enabled genomic studies across a wide range of non-model organisms (Hotaling et al., 2021).

For non-model organism research, this technological shift was transformative. Prior to NGS, developing even basic genetic resources required substantial technical expertise, specialized infrastructure, and funding, restricting genetic studies largely to well-established model organisms or species of economic relevance (Ekblom and Galindo, 2011). The widespread adoption of short- and, more recently, long-read sequencing technologies, often combined in hybrid strategies, now enables high-quality genome assemblies even for species with complex genome architectures, including high heterozygosity, polyploidy, or extensive repetitive content (Zimin et al., 2017; Appels et al., 2018; Gerdol et al., 2020).

**Figure 2 -:**
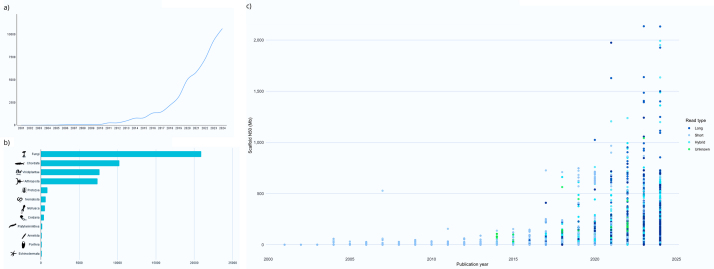
Overview of eukaryotic genome assemblies deposited in NCBI (data from NCBI Genome Database, accessed October 2025). (a) Number of eukaryotic genomes available in NCBI per year, shown up to 2024 since 2025 is still ongoing. (b) Distribution of eukaryotic genome assemblies across major taxonomic groups. (c) Genome assembly quality, represented by the Scaffold N50 (Mb), a metric that reflects the contiguity of genome assemblies. “Hybrid” indicates sequencing projects that combine both long- and short-read technologies.

These developments are reflected in the rapid expansion and diversification of genome assemblies across the tree of life (Figure 2). Large-scale international initiatives, such as the Earth BioGenome Project, the Vertebrate Genomes Project, i5K, and the 10,000 Plant Genomes Project, have greatly increased the number of available eukaryotic genomes (i5K Consortium, 2013; Cheng et al., 2018; Rhie et al., 2021; Blaxter et al., 2025). As shown in Figure 2 a , genome availability has grown exponentially in recent years, while Figure 2 b highlights persistent taxonomic biases, with many invertebrate lineages remaining underrepresented despite their ecological importance (Lopez et al., 2019). In parallel, the increasing use of long-read and hybrid sequencing strategies has substantially improved genome assembly contiguity and quality (Figure 2 c ).

Finally, the expansion of genomic studies to non-model organisms has been critically supported by advances in bioinformatic tools and computational resources. Sophisticated pipelines for genome assembly, annotation, and comparative analysis are now essential components of evolutionary genomics, enabling the integration and interpretation of the large-scale datasets generated by modern sequencing efforts (Muir et al., 2016).

## Beyond sequencing genomes

Beyond sequencing technologies, advances in biotechnology have further accelerated research on non-model organisms. Since its introduction in 2012 (Jinek et al., 2012), the CRISPR-cas9 system has become a widely adopted genome-editing tool for functional genetic studies. Its key conceptual innovation lies in target specificity being dictated by a small guide RNA rather than by locus-specific protein engineering, which was required for earlier approaches such as zinc-finger nucleases and TALENs (Gaj et al., 2013; Li et al., 2023 b). These earlier methods constrained editing largely to well-established model organisms, whereas CRISPR-Cas9 substantially simplified workflows, increased throughput, and enabled broader adoption across both model and non-model species (Pacesa et al., 2024; Zhang et al., 2025).

In parallel, advances in multi-omics have transformed molecular characterization of both mutant and wild-type organisms, with several recent works integrating such approaches on the investigation of gene function (Li et al., 2022; Ding et al., 2023; Ni et al., 2025). Hence, integrating genomics, transcriptomics, epigenomics, proteomics and metabolomics allows comprehensive assessment of gene function and regulation. Transcriptomic analyses have become routine, with *bulk* RNA-seq remaining the standard for isoform-level analyses and alternative splicing, while single-cell RNA-seq has enabled transcriptome-wide profiling at cellular resolution (Thind et al., 2021; Li *et al*., 2023b; Ament et al., 2025).

Epigenomic assays further expanded functional inference by profiling DNA methylation, histone modifications, chromatin accessibility, and three-dimensional genome organization, revealing mechanisms of gene regulation, cell differentiation states and environmental responses that are not captured by either genomic or transcriptomic analyses (Shema et al., 2019; Schübeler, 2015). Techniques such as ChIP-seq (Johnson et al., 2007) and ATAC-seq (Buenrostro et al., 2013) replaced earlier chromatin-mapping approaches that required extensive sample processing and high input material, collectively shortening experimental timelines, improving reproducibility, and reducing costs (reviewed in Shema *et al.*, 2019; Khodadadi et al., 2021).

Finally, the integration of proteomics and metabolomics provides complementary functional validation of genomic predictions by measuring downstream molecular activity, thereby linking genotype to molecular phenotype and cellular function (Gutierrez-Reyes et al., 2024). Advances in automated sample preparation and analytical pipelines have increased throughput and accessibility of these approaches (Fu et al., 2023; Ghafari and Sleno, 2024; Sanchez-Avila et al., 2025). As a result, multi-omic frameworks are now widely applied to developmental, adaptive, and evolutionary studies in both, model and non-model organisms, including investigations of convergent evolution (Drabeck et al., 2025) and experimental adaptation under resource limitation (Tamminen et al., 2018).

## New insights into evolutionary processes

The technological advances and the rapid expansion of genome sequencing since the early 2000s (Figure 2a ) discussed earlier have enabled researchers to address fundamental questions in evolutionary biology that were previously inaccessible, allowing us to gain new perspectives on evolutionary processes. In this section, we highlight some areas where studies of non-model organisms have particularly enriched our understanding of evolution.

### Convergent evolution: Molecular underpinnings of repeated adaptations 

The genomic era has transformed our understanding of how similar traits evolve independently across the tree of life. What once appeared as straightforward cases of convergent evolution, such as the textbook examples of bat and bird wings, or the camera eyes of vertebrates and cephalopods, are now known to be complex molecular phenomena occurring across multiple levels of biological organization (Hoitinga and Birkeland, 2025). The expansion of genetic studies beyond traditional model organisms has been crucial in unveiling this complexity, demonstrating that the simple morphological framework of “similar traits in distantly related species” fails to capture the intricated mechanisms underlying repeated evolutionary solutions (Sackton and Clark, 2019).

Recent frameworks reconceptualize convergent and parallel evolution as part of a *repeated evolution* continuum rather than discrete categories (Nery et al., 2026). This perspective acknowledges that similar phenotypes can arise from changes at multiple levels of biological organization (Storz, 2016). In this context, non-model organisms provide natural experiments that would be impossible to replicate in laboratory settings.

For example, genomic studies across diverse lineages have revealed sequence-level convergence through identical amino acid substitutions, as seen in rhodopsin adaptations across fish species (Hill et al., 2019). Gene-level functional convergence occurs when different genes achieve similar outcomes, exemplified by Hox gene evolution in aquatic mammals, where different paralogous genes were targeted by selection but had equivalent functions in body shaping (Nery et al., 2016). Regulatory convergence emerges through changes in gene expression patterns, as demonstrated in flightless birds, where thousands of conserved non-coding elements linked to limb development show accelerated evolution (Sackton et al., 2019). Pathway-level convergence involves modifications in equivalent biological pathways through different molecular routes, most prevalent in distantly related taxa adapting to similar environments (Witt and Huerta-Sánchez, 2019). 

The level at which convergence occurs correlates with phylogenetic distance: closely related species typically converge through identical or similar genetic changes, while distantly related taxa achieve similar adaptations through pathway-level modifications (Bridgham, 2016). However, this pattern shows exceptions, as even closely related species can achieve convergence through different genes with similar functions rather than identical changes, as seen in the evolution of toxin resistance in different snake populations (Morales et al., 2024).

These insights from non-model organisms have great implications for our understanding of evolutionary predictability and constraint. The multi-level nature of convergent evolution reveals that while natural selection may repeatedly favor similar phenotypic solutions to environmental challenges, the molecular paths to these solutions are more diverse than previously imagined (Blount et al., 2018). The prevalence of regulatory and pathway-level convergence, particularly in complex traits, suggests that evolution has more degrees of freedom than implied by model organism studies, which often emphasized sequence-level changes (Stern and Orgogozo, 2008; Martin and Orgogozo, 2013). Furthermore, the discovery that convergence can occur through different mechanisms even in closely related species challenges our ability to predict evolutionary outcomes based solely on phylogenetic relationships (Stern, 2013).

The expanded view of convergent evolution, revealed through comparative genomics of diverse organisms, fundamentally alters our perspective on adaptation. Rather than being constrained to a limited set of molecular solutions, multiple independent lineages have arrived at similar phenotypic endpoints through distinct molecular routes (Sackton and Clark, 2019). As we continue to explore genomic diversity across the tree of life, we will likely discover even more mechanisms underlying convergent phenotypes, further enriching our understanding of how predictable and repeatable evolutionary processes truly are (e.g. Wilhoit et al., 2025).

### Molecular mechanisms of speciation

By expanding genomic studies beyond traditional models, researchers have revealed speciation as a heterogeneous process where different genomic regions differentiate at varying rates due to the interplay of selection, gene flow, and genomic architecture, providing a more nuanced view than previously possible through studies of *Drosophila* alone (Seehausen et al., 2014; Wolf and Ellegren, 2017)

The concept of “genomic islands of differentiation” has been refined through studies of diverse taxa. Initial work in *Anopheles* mosquitoes, sunflowers (*Helianthus*), and sticklebacks (*Gasterosteus*) suggested that differentiation was concentrated in specific genomic regions resistant to gene flow (Turner et al., 2005; Jones et al., 2012; Renaut et al., 2013). However, broader taxonomic sampling revealed that these patterns vary dramatically across species. In some cases, such as Darwin’s finches, differentiation is indeed concentrated in a few large-effect loci controlling key adaptive traits (Lamichhaney et al., 2015). In others, like *Heliconius* butterflies, differentiation is distributed across many small-effect loci throughout the genome, while in some cases also including large-effect genes associated with traits such as mimicry, underscoring that no single genomic architecture universally explains speciation across taxa (Edelman et al., 2019).

Structural variants have emerged as major drivers of speciation in non-model systems (Mérot et al., 2020). Chromosomal inversions suppress recombination and maintain adaptive gene complexes, as documented in *Mimulus* monkeyflowers (Fishman et al., 2013) and Atlantic cod (Berg et al., 2016). These inversions can capture multiple adaptive alleles and facilitate rapid adaptation despite gene flow, challenging the traditional view that geographic isolation is necessary for speciation (Faria et al., 2019).

The role of hybridization and introgression in speciation has also been revolutionized by genomic studies of non-model organisms. Rather than being an evolutionary dead-end, hybridization is now recognized as a creative force in evolution (Abbott et al., 2013; Taylor and Larson, 2019). Adaptive introgression has been documented in wolves and dogs (vonHoldt et al., 2016), *Populus* trees (Suarez-Gonzalez et al., 2018), and notably in humans, where Neanderthal and Denisovan alleles contribute to high-altitude adaptation and immune function (Racimo et al., 2015). Hybrid speciation, once thought rare, has been documented in *Helianthus* sunflowers (Rieseberg et al., 2003), Lake Victoria cichlids (Meier et al., 2017), and sparrows (Hermansen et al., 2014).

Genomic studies of non-model organisms revealed speciation as a genomically heterogeneous process where reproductive isolation emerges through diverse mechanisms operating at multiple scales, from point mutations to chromosomal rearrangements (Ravinet et al., 2017; Wolf and Ellegren, 2017). The widespread occurrence of hybridization and introgression as creative forces challenges the traditional view of speciation as a strictly bifurcating process and highlights how genetic exchange between lineages can fuel adaptive evolution (Edelman and Mallet, 2021). Most importantly, the discovery that genome architecture itself evolves to facilitate speciation, through inversions, supergenes, and other structural variants, demonstrates that the genomic substrate of evolution is highly dynamic and that this dynamism becomes more evident when evolutionary inference extends beyond the limited set of classical model organisms traditionally studied (Mérot et al., 2020).

### Dynamics of genome evolution

Studies of non-model organisms have revealed extensive variation in genome size, structure, and content that was unimaginable from model organism studies alone. The C-value paradox, the lack of correlation between genome size and organismal complexity, has been illuminated through comparative genomics across diverse taxa (Elliott and Gregory, 2015; Blommaert, 2020).

The *Paris japonica* genome, at 150 Gb (Pellicer et al., 2018), vastly exceeds the size of the carnivorous bladderwort *Utricularia gibba*, which maintains full plant complexity with only 82 Mb (Ibarra-Laclette et al., 2013). These extremes have revealed that genome size evolution is driven by the balance between DNA gain through transposable element activity and DNA loss through deletion bias (Kapusta et al., 2017). In the japanese pufferfish (*Fugu rubripes*), the compact genomes result from strong deletion bias removing non-essential sequences (Aparicio et al., 2002), while in lungfish, relaxed selection likely allowed massive transposon accumulation (Wang et al., 2021).

Polyploidy and whole-genome duplications (WGDs) have been revealed as major evolutionary drivers across eukaryotes. While rare in model organisms except *Arabidopsis*, polyploidy is prevalent in non-model systems. Teleost fishes experienced a lineage-specific whole-genome duplication in addition to the two ancient genome duplications shared with other vertebrates, contributing to the diversification of gene repertoires and evolutionary trajectories within this group (Glasauer and Neuhauss, 2014), salmonids underwent additional recent duplications enabling cold adaptation (Lien et al., 2016), and polyploidy is widespread in plants, with multiple independent origins in crops like wheat, cotton, and strawberry (Jiao et al., 2011; Zhang et al., 2015; Edger et al., 2019). 

Horizontal gene transfer (HGT), once thought to occur exclusively in prokaryotes, is now recognized as a significant driver of eukaryotic evolution (Keeling and Palmer, 2008). Studies on non-model organisms have revealed several instances of HGT, such as plant-parasitic nematodes acquiring cellulase genes from bacteria (Danchin et al., 2010) and aphids obtaining carotenoid biosynthesis genes from fungi (Moran and Jarvik, 2010). Bdelloid rotifers exhibit particularly extensive HGT, with approximately 8% of their genes derived from bacteria, fungi, and plants, likely facilitated by repeated desiccation-rehydration cycles (Nowell et al., 2018). The integration of foreign DNA, which provides raw material for evolutionary innovation and challenges traditional notions of species boundaries and vertical inheritance, appears far more common than once believed (Soucy et al., 2015).

Transposable elements (TEs), once dismissed as “junk DNA,” have emerged as major architects of genome evolution in non-model organisms (Chuong et al., 2017). While TEs comprise only ~3% of the *Drosophila* genome and are largely inactive in *C. elegans*, they constitute 45% of the human genome and up to 85% of plant genomes like maize and wheat (Wicker et al., 2007; Schnable et al., 2009). Studies of non-model organisms revealed extraordinary TE diversity and activity: the axolotl genome (32 Gb) is dominated by long terminal repeat retrotransposons that expanded recently (Nowoshilow et al., 2018), while the octopus genome shows massive expansion of transposon families specifically in neural tissues, potentially contributing to behavioral complexity (Albertin et al., 2015). In bats, DNA transposons remain active and have been horizontally transferred between species, challenging the view that DNA transposons are inactive in mammals (Ray et al., 2008; Pagán et al., 2012). Beyond genome expansion, TEs have been powerful drivers of regulatory innovation. The massive expansion of KRAB zinc finger genes in tetrapods, which evolved to repress TEs, illustrates how TE-host interactions can generate species-specific regulatory networks (Imbeault et al., 2017; Helleboid et al., 2019).

The discovery of pervasive non-coding RNA (ncRNA) transcription across diverse taxa has also reshaped our understanding of genome functionality by revealing how widespread, variable, and evolutionarily dynamic these regulatory layers are across lineages. Long non-coding RNAs (lncRNAs), which occupy a substantial fraction of complex eukaryotic genomes and are largely transcribed by RNA polymerase II, evolve rapidly and display highly cell-type- and lineage-specific expression patterns. Despite limited sequence conservation, many lncRNAs exhibit conserved structures and regulatory functions, underscoring their central roles in chromatin organization, development, and cellular differentiation (Mattick et al., 2023). The coelacanth genome revealed ancient conserved lncRNAs predating the tetrapod transition (Amemiya et al., 2013), while comparison across 17 vertebrate species showed that only 2-5% of lncRNAs are deeply conserved, suggesting continuous evolutionary innovation (Hezroni et al., 2015). Small RNA pathways also show remarkable diversity: plant microRNAs differ fundamentally from animal microRNAs in biogenesis and targeting (Axtell, 2013), ciliate genomes encode thousands of small RNAs guiding genome rearrangement (Hamilton et al., 2016), and piwi-interacting RNAs (piRNAs) have independently evolved in arthropods, nematodes, and vertebrates to control transposons through distinct mechanisms (Ozata et al., 2019). 

The genomic diversity revealed by non-model organisms has altered the notion of a universal genome architecture, yet much of this progress still comes from vertebrates and plants in particular, where protocols and reference resources are well established (Figure 2 b ). Importantly, the overwhelming majority of microbial diversity remains inaccessible to standardized experimental approaches underscoring how much of life’s diversity remains unexplored and reinforcing the need for broader taxonomic sampling to understand how biodiversity is generated and maintained (Steen et al., 2019). 

### Case studies in aquatic mammals

**Figure 3 -:**
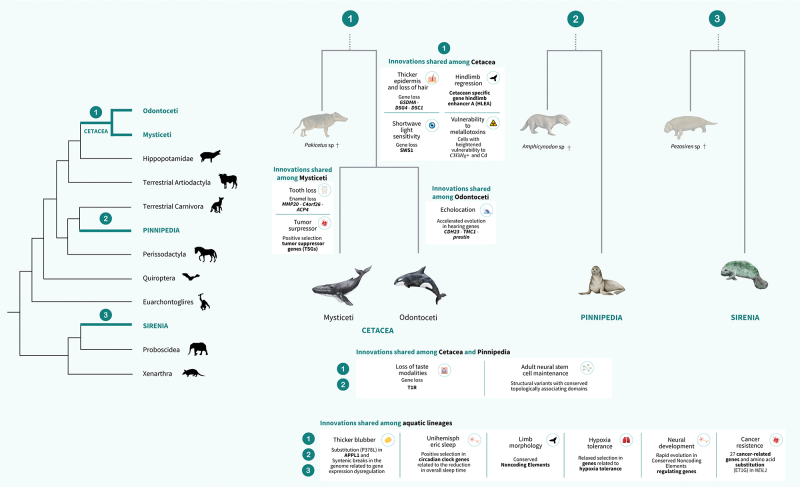
Aquatic mammals evolution. The diagram presents three distant aquatic mammal groups, illustrating convergent and parallel evolution. On the left, we present a mammalian phylogenetic tree as a reference of the phylogenetic relationship between the three aquatic mammal lineages. Numbers indicate the aquatic mammal groups: 1. Cetacea; 2. Pinnipedia; 3. Sirenia. Boxes highlight genomic evidence for aquatic adaptations. ✝ represents an extinct order species.

For a long time, our understanding of aquatic mammals’ evolution was based primarily on a fossil perspective. Over the last three decades, molecular studies have significantly broadened our knowledge of their evolutionary history, especially regarding their transition from land to water. As non-model organisms, aquatic mammals can be a valuable natural experiment to understand evolutionary processes: pinnipeds (seals, sea lions, walruses), sirenians (dugong, manatees), and cetaceans (dolphins, whales) have independent evolutionary origins, yet they convergently evolved similar solutions to the challenges of aquatic life, including locomotion, thermoregulation, sensory perception, communication, sleep patterns, and hypoxia tolerance, among others (McGowen et al., 2014, 2020; Policarpo et al., 2024; Selleghin-Veiga et al., 2024; Xu et al., 2025; Yin et al., 2025) (Figure 3).

Within the last decades’ advancements comparative genomics allowed us to better evaluate convergent phenotypes. For instance, by comparing the genomes of pinnipeds, sirenians, and cetaceans it was possible to understand how chemoreceptor losses are related to dietary adaptation. Carnivorous marine mammals (cetaceans and pinnipeds) have lost all T1R taste genes, while herbivorous sirenians have maintained the T1R genes (Policarpo et al., 2024). Another example is the convergent physiological adaptation in the unihemispheric sleep (USWS), which enables cetaceans, manatees, and walruses to address the challenges of sleeping in water while still requiring air to breathe (Lyamin and Siegel, 2019). This adaptation is likely linked to positive selection in circadian clock genes (*clock* and *bmal1*). It was further tested in cell-based functional assays, which demonstrated that cetacean-specific mutations enhanced the transcriptional activation activity of the *clocka* and *bmal1a* genes, resulting in a reduction in overall sleep time (Yin et al., 2025). 

Cetaceans are also an interesting group for studying divergent evolution, where closely related lineages evolve distinct solutions to ecological challenges. An example is the divergence between odontocetes and mysticetes, which have distinct feeding strategies (Fordyce, 2001). While odontocetes have teeth and rely on echolocation for hunting, the whales have a filter feeding baleen (Bannister, 2002). The divergence of these phenotypes offers a framework for understanding the environmental drivers and genetic mechanisms of cetacean speciation. For odontocetes, echolocation was a crucial development for their feeding habits, enabling adaptation to diverse environments (Au, 2002). Other comparative genomic analysis revealed accelerated evolution in hearing genes (CDH23, TMC1, prestin) among coastal and riverine species, likely due to selective pressures on sonar propagation in shallow habitats (Magpali et al., 2024). On the other hand, mysticetes lost the tooth enamel proteins, which may be related to the evolution of the baleen (Springer et al., 2019). The cetaceans lineage-specific adaptation extends beyond feeding morphology to physiology, which becomes evident in traits such as cancer resistance. Cetaceans exhibit a rapid turnover rate of tumor suppressor genes (TSGs), approximately 2.4 times faster in cetaceans than in other mammals (Tejada-Martinez et al., 2021). However, most duplication events and positively selected genes were in the lineage of mysticetes, suggesting that they have evolved additional anticancer mechanisms (Keane et al., 2015; Tollis et al., 2019; Nery et al., 2022). 

In summary, aquatic mammals studies exemplify the importance of non-model organisms in evolutionary biology. The research with aquatic mammals is not limited to how convergence can arrive at similar phenotypes via different genetic routes, or how closely related lineages can evolve distinct traits (McGowen et al., 2014, 2020; Policarpo et al., 2024; Selleghin-Veiga et al., 2024; Xu et al., 2025). It expands our knowledge of fundamental rules of evolution and the role of natural selection, leading to a better understanding of genetics, development and physiology (Lyamin and Siegel, 2019; Nery et al., 2022; Yin et al., 2025).

## Conclusion and future perspectives 

The expansion of genomics from a narrow focus on a few model organisms to the inclusion of diverse species across the tree of life has reshaped evolutionary biology. Studies in non-model organisms have revealed that the molecular mechanisms underlying adaptation, speciation, and genome evolution are far more dynamic, heterogeneous, and creative than previously envisioned. This transformation reflects how genetics has matured as a field. We have moved from seeking simple universal rules to appreciating the complex interplay between general principles and lineage-specific innovations. Non-model organisms have thus redefined the boundaries of what constitutes a “model,” proving that every lineage carries unique insights into the mechanisms of evolution.

The future of the evolutionary genomics field lies in the continued integration of non-model organisms into large-scale comparative frameworks. As sequencing technologies advance toward real-time, high-throughput, and low-cost applications, the genomic representation of Earth’s biodiversity is expected to expand dramatically. In parallel, progress in single-cell sequencing is revealing an unexpected level of cellular diversity across organisms (Arendt et al., 2016), while pangenomic approaches are demonstrating that reliance on a single reference genome overlooks much of the genetic variation within species (Computational Pan Genomics Consortium, 2018). Moreover, advances in ancient DNA technologies now enable the reconstruction of evolutionary trajectories across thousands of years, bridging modern genomics with the historical dynamics of populations and ecosystems. These are only a few of the advances that now allow us to explore non-model species at unprecedented depth, providing powerful means to uncover the molecular mechanisms that drive evolution.

One major frontier is the incorporation of multi-omics approaches, combining genomics, transcriptomics, epigenomics, proteomics, and metabolomics, to uncover how molecular and regulatory layers interact to shape phenotypic diversity. This integration will allow researchers to connect genotype-to-phenotype links in ecologically and evolutionarily meaningful contexts.

Functional validation of genomic predictions is becoming possible for more species through experimental tools such as CRISPR-Cas9, induced pluripotent stem cells, and organoid systems derived from non-model species, further bridging the gap between sequence data and organismal biology (Ford et al., 2019). 

Yet challenges remain substantial. Annotating regulatory regions is still difficult without extensive functional data, and most genetic tools are still designed for a handful of species. Data integration across studies is messy due to inconsistent standards. Perhaps most importantly, we are still missing many branches of the tree of life, as marine invertebrates, protists, and the microbiomes associated with most organisms remain largely unexplored genomically (Schoenle et al., 2025). Each of these groups likely contains innovations that could transform our understanding of what is possible in molecular evolutionary biology.

The continued expansion of access to sequencing and analysis tools ensures that the next decades will witness an even broader participation of global research communities in evolutionary discovery. Together, these efforts will deepen our understanding of how genomes evolve, how biodiversity arises and persists, and how life continuously reinvents itself in response to ecological and environmental challenges. In sum, from few to many represents more than technological progress. It is a recognition that to really understand evolution, its shared principles, and its creativity, we need to embrace the full diversity of life.

## Data Availability

The entire dataset supporting the results of this study was published in the article itself.
